# CT/MRI影像融合对肺癌脑转移放射治疗靶区勾画的影响

**DOI:** 10.3779/j.issn.1009-3419.2012.08.05

**Published:** 2012-08-20

**Authors:** 洋 李, 香兰 李, 业伟 王, 洋 周

**Affiliations:** 1 150081 哈尔滨，哈尔滨医科大学附属第三医院放疗科 Department of Radiotherapy, the Third Affiliated Hospital of Harbin Medical University, Harbin 150081, China; 2 150081 哈尔滨，哈尔滨医科大学附属第三医院放疗科物理室 Department of Radiation Physicst, the Third Affiliated Hospital of Harbin Medical University, Harbin 150081, China; 3 150081 哈尔滨，哈尔滨医科大学附属第三医院影像中心 Medical Image Center, the Third Affiliated Hospital of Harbin Medical University, Harbin 150081, China

**Keywords:** 肺肿瘤, 脑转移, CT/MRI影像融合, 靶区勾画, Lung neoplasms, Brain metastasis, CT/MR image registration, Target delineation

## Abstract

**背景与目的:**

脑转移瘤靶区勾画的准确性一直是放射治疗的关键，CT/MRI融合技术提供了可行的方法，本研究旨在探讨CT/MRI图像融合技术在肺癌脑转移靶区勾画中的作用。

**方法:**

将31例肺癌脑转移患者的增强CT和MRI图像传送至图像处理工作站，分别在CT和CT/MRI融合图像上勾画GTV，比较勾画后的GTV体积，分析最大平均误差及瘤周水肿对靶区勾画的影响。

**结果:**

CT/MRI融合图像上勾画的GTV明显小于CT图像上勾画的GTV；瘤周水肿对靶区勾画存在明显影响。

**结论:**

CT/MRI图像融合技术可以提高肺癌脑转移靶区勾画的准确性。

## Abstract (en)

**Background and objective:**

Accurate target delineation in radiation therapy is a key component of the treatment regimen for brain metastasis for which CT/MRI fusion technology provides a feasible method. The aim of this study is to explore the role of CT/MRI image registration in target delineation for lung cancer with brain metastasis.

**Methods:**

The image data of 31 patients were processed using Oncentra MasterPlan. The GTVs were delineated on CT and CT/MRI images, and their differences were compared to analyze the impact of the maximum average error and tumor edema on target delineation.

**Results:**

The GTVs delineated on CT/MRI images were markedly smaller than those delineated on CT images. Target delineation was clearly influenced by edema.

**Conclusion:**

The technology of CT/MRI image registration can improve the accuracy of target delineation for lung cancer with brain metastasis.

肺癌为脑转移最常见的原发肿瘤，约占全部脑转移瘤的30%-40%且为升高趋势^[1]^，自然中位生存期仅为1个月-2个月^[2]^。计算机断层扫描（computed tomography, CT）图像一直是放疗中靶区和危及器官勾画的最佳图像资源，但由于对软组织结构区分的能力差，肿瘤边界模糊，即使采用增强CT仍有部分病灶显示不清或不显示，脑转移瘤常多发且伴有水肿，使得通过CT及参考常规的核磁共振成像（magnetic resonance imaging, MRI）胶片勾画靶区时很难界定肿瘤区（gross tumor volume, GTV）边界，往往造成靶区勾画过大、靶区勾画不确定。本研究通过对比影像融合前后勾画的靶区，探讨CT/MRI图像融合技术在肺癌脑转移靶区勾画中的作用。

## 1 资料与方法

### 1.1 研究对象

本组病例为2011年1月-2012年5月哈尔滨医科大学附属第三医院收治的肺癌脑转移患者，共31例，均经病理证实。其中男性15例，女性16例，年龄48岁-73岁；鳞癌8例，腺癌16例，小细胞癌7例；单发23例，多发8例，其中幕上25例，幕下5例，幕上及幕下转移1例。对于伴有明显颅高压症状者扫描前均行降颅压药物治疗，且CT及MRI扫描间隔尽量缩短。对于CT和MRI均呈阴性的病例，如脑膜转移，因融合技术优势不明显，且脑膜转移多采用全脑照射，本研究并未纳入相关病例。

### 1.2 CT/MRI检查

全部病例均在仰卧位予热塑面罩固定后行横断面CT增强扫描，采用菲利浦大孔径模拟定位机（Phylips Brilliance Big Bore），图像轴面扫描基线采取眶耳线（外眦至外耳道连线），直至颅顶骨，厚度为5 mm，冠状面扫描基线为冠状线垂直眶耳线，扫描层厚为5 mm。所有病例在CT扫描前后3天内均接受MRI扫描，MRI采用Phillips Achieva 1.5T机，扫描时均采用头颈联合线圈。由于受MRI机孔径限制，扫描时未能予热塑面罩固定，扫描范围同CT扫描范围，层厚5 mm。

### 1.3 图像融合方法及靶区勾画

扫描后将CT及MRI图像经局域网传输至治疗计划系统（VARIN Eclipse version 10.0），本系统可根据CT图像及MRI图像分别重建三维图像，采用Point Match法，选择基底动脉、晶体、视神经、脑干等作为参考点进行融合，融合最大误差、平均误差均控制在2 mm以内，完成后由一位高年资放疗科医师分别对每例患者增强CT图像及CT/MRI融合后的图像进行靶区勾画。红色为GTVCT，绿色为GTVCT/MRI，利用VARIN治疗计划系统上靶区体积计算功能分别计算出各例患者基于CT/MRI融合图像及增强CT图像所勾画的GTV体积并计算比值，若比值介于0.9-1.1，为两种靶区勾画方法体积接近；比值≤0.9或≥1.1均为两种靶区勾画方法差异明显。最大平均误差用两组GTV边界相差最大值的均数来表示。

### 1.4 瘤周水肿情况

水肿分度：无水肿，肿瘤周围无明确水肿带；轻度，水肿范围 < 肿瘤最大径的1/2；中度，水肿范围在肿瘤最大径的1/2至肿瘤最大径之间；重度，水肿范围 > 肿瘤最大径。如果为多发脑转移的患者，以水肿最严重的病灶统计。本组31例患者中，无水肿5例，轻度5例，中度9例，重度12例。

### 1.5 统计方法

数据处理采用SPSS 13.0统计软件，GTV体积比较采用*t*检验，多组之间比较采用方差分析，两两比较采用*LSD*检验。以*P* < 0.05为差异有统计学意义。

## 2 结果

### 2.1 GTV体积比较

增强CT靶区的平均体积为23.17 cm^3^，明显大于CT/MRI融合靶区的11.15 cm^3^，CT图像上勾画的GTV平均值为（23.17±3.46）cm^3^，CT/MRI融合图像上勾画的GTV平均值为（11.15±1.93）cm^3^，两者差异有统计学意义（*t*=3.021, *P*=0.004）。基于CT/MRI融合图像与增强CT图像所分别勾画的GTV体积比较见表 1。

**1:** CT/MRI融合图像与增强CT图像勾画的GTV体积比较 The comparison between GTV volume of CT/MRI image registration and enhanced CT image

GTVCT/MRI/GTVCT	Cases	Proportion (%)
≤0.9	26	0.84
0.9-1.1	4	0.13
≥1.1	1	0.03

### 2.2 瘤周水肿对靶区勾画的影响

无水肿的最大平均误差为（0.34±0.02）cm，因1例GTVCT/MRI大于GTVCT，考虑为CT靶区勾画偏小，不在比较范围而将其排除。瘤周水肿对于靶区勾画影响较大，瘤周水肿程度越大，靶区勾画准确度越低（表 2）。多组之间比较差异有统计学意义（*F*=3.572, *P*=0.041），两两之间比较显示无水肿靶区勾画的最大平均误差与中度水肿和重度水肿靶区勾画的最大平均误差存在统计学差异（*P*=0.015, *P*=0.012）。

**2:** 转移灶不同水肿程度的靶区勾画最大平均误差比较 The comparison among maximum average errors of various degrees of tumor edema

Degree of edema	Cases	Maximum average errors (cm)
None	4	0.34±0.02
Light	5	0.68±0.25
Moderate	9	0.95±0.13
Severe	12	1.02±0.11

## 3 讨论

肺癌是脑转移中最常见的原发肿瘤，约占全部脑转移瘤的30%-40%^[1]^，CT平扫多表现为颅内单发或多发的等密度、低密度和高密度，瘤灶周围明显水肿是转移瘤的一个明显特征，呈低密度区，水肿常累及白质，而较少累及灰质，典型的水肿呈指状分布，但部分脑转移瘤CT平扫呈等密度，而病灶周围又无脑水肿，因此很难发现（图 1）。

**1:**
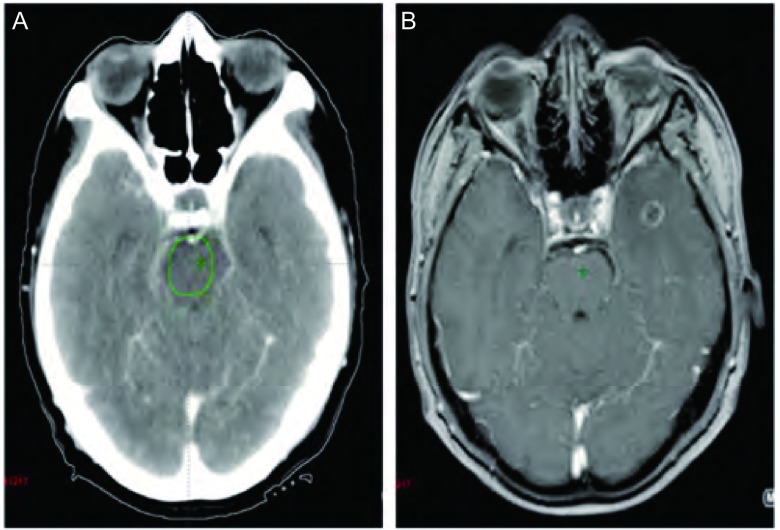
增强CT图像（A）和MRI图像（B） Enhanced CT image (A) and MRI image (B)

MRI成像取决于物质的质子密度，对软组织特别是对浸润性肿瘤比较敏感，图像边界清晰，较CT影像能够更精确地描述肿块大小，并减少观察之间和观察者自身对肿瘤范围理解上的差异^[3]^，MRI对于脑转移瘤的诊断和GTV的确定明显优于CT，但由于MRI非线性的磁场和体内不同组织的磁化系数不同，存在着图像的失真问题，更重要的是MRI不能提供剂量所需的诸如电子密度、阻止本领比等参数，加上扫描时间长和缺少固定装置，所以，MRI的图像一般不能用于治疗计划系统。

医学图像融合技术是20世纪90年代出现的一种图像后处理方法，充分发挥了MRI和CT各自的优势，在一定程度上弥补了MRI变形、失真的不足，而对肿瘤边界的显示则更加清晰。目前该技术在鼻咽癌、脑胶质瘤中研究较多，尚未见应用于脑转移瘤中的报道。张蕾等^[4]^收集了19例脑胶质瘤患者进行了术前术后CT与MRI的融合，发现靶区的勾画单靠CT或者MRI都没有两者融合的效果好，显示CT/MRI融合图像明显优于单独MRI图像。李丹明等^[5]^也得出了相似结论。本组病例中CT图像上勾画的GTV平均值（23.17±3.46）cm^3^，CT/MRI融合图像上勾画的GTV平均值（11.15±1.93）cm^3^，两者差异有统计学意义，CT图像上勾画的GTV明显大于CT/MRI融合图像上勾画的GTV，这主要是因为：①CT图像无法完整清晰地显示肿瘤边界；②为了不遗漏靶区，主观上外扩了靶区范围，而CT/MRI融合后图像具备了两种影像技术的优势，因此缩小了靶区范围。本研究重点探讨融合前后靶区范围的比较，因此对亚临床的转移和转移灶的亚临床浸润即CTV并未做比较。在图像融合过程中，笔者应用治疗计划系统中的融合软件包进行图像配准和融合处理。没有采用外部定位标记，而选取颅脑内部标志点作为参考点，主要因为颅脑内的一些解剖结构位置固定并且容易在CT和MRI图像中识别，如大脑基底动脉、视交叉点、晶体、视神经等，均可在CT和MRI图像中清晰显示，且融合结果的优劣由一位影像医师及一位放疗医师共同判定。采用体表标记法，虽然可以为融合提供更好的位置标准，但在患者活动及行扫描时均存在标记物发生移动的可能，即使很小的位移也会导致融合时出现较大的误差。

本组病例中，无水肿5例，轻度水肿5例，中度水肿9例，重度水肿12例。其中无水肿患者的最大平均误差为（0.34±0.02）cm（因1例GTVCT/MRI大于GTVCT，考虑为CT靶区勾画偏小，不在比较范围而将其排除），中度和重度水肿患者的最大平均误差分别为（0.95±0.13）cm、（1.02±0.11）cm，无水肿患者的最大平均误差与中、重度水肿患者的最大平均误差有统计学差异（*P* < 0.05）。由此可见靶区勾画的误差大小与脑水肿程度成正比，即脑水肿程度越大，靶区勾画越不准确（图 2）。主要因为瘤周水肿虽然可以判定转移瘤的大致位置，但由于边界不清，且瘤体大小常与瘤周水肿程度不成比例，即表现为“小病灶，大水肿”，为避免遗漏靶区，往往造成靶区勾画过大，本组重度水肿患者的靶区勾画最大平均误差达到1 cm以上。另外对于幕下等部位的瘤灶，虽然受水肿影响很小，但由于缺乏密度对比，病灶边缘亦显示不理想，无法满足对于靶区高精度的要求。从图 3可见增强CT对于小脑病灶显示不清，CT/MRI融合后的图像则可以较好地显示瘤灶边界，两者最大误差达到1.34 cm。因此建议对于水肿明显或者增强CT显示不清的病灶，尽量应用图像融合技术，以减少误差。

**2:**
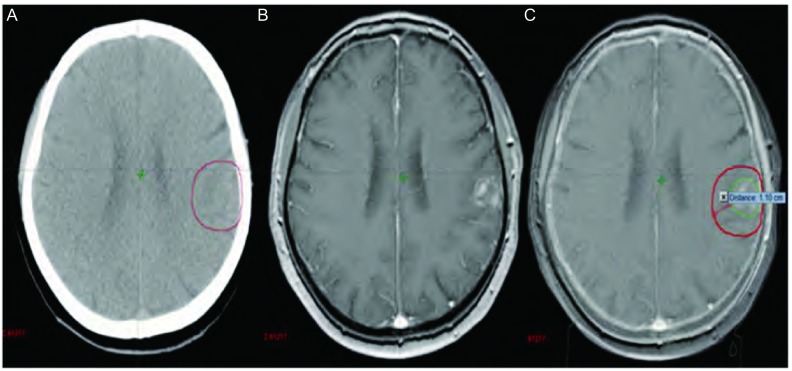
幕上转移灶（A：增强CT图像；B：MRI图像；C：CT/MRI融合图像）。 Supratentorial metastasis. (A: Enhanced CT image; B: MRI image; C: CT/MRI fusion image).

**3:**
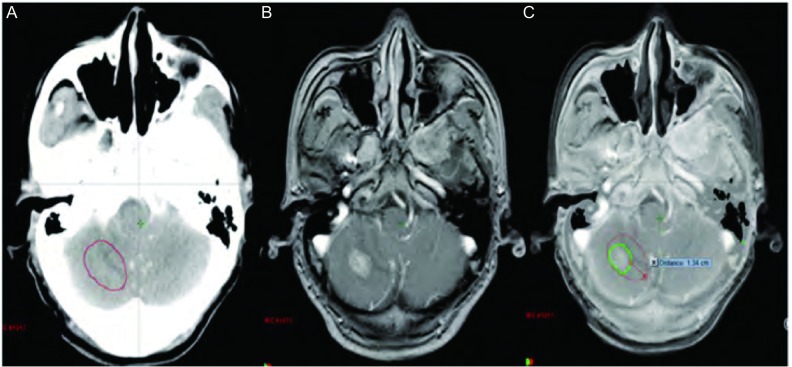
幕下转移灶（A：增强CT图像；B：MRI图像；C：CT/MRI融合图像）。 Infratentorial metastasis (A: Enhanced CT image; B: MRI image; C: CT/MRI fusion image).

多发脑转移在肺癌中占有一定比例，本组病例为25.8%（8/31），转移灶多呈散发且体积较小，瘤周水肿常不明显，因此CT平扫很难完全显示。CT/MRI图像融合技术具备了MRI的成像特点，对于多发小病灶特别是微小病灶和后颅窝病变具有明显优势。本组病例中单发23例，多发8例，因两者病例数相差较多，未作统计学比较，但笔者发现通过影像融合技术可以更准确地判定病灶位置，大大降低了在CT图像上寻找病灶的难度，减少了主观误差（图 4）。

**4:**
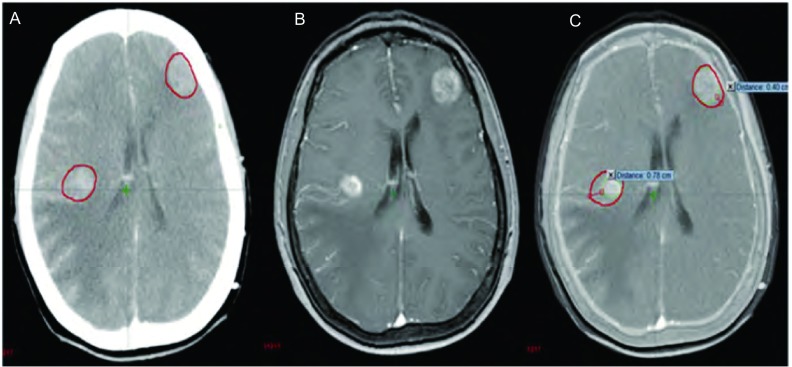
多发转移灶（A：增强CT图像；B：MRI图像；C：CT/MRI融合图像）。图 2-图 4中A、B、C分别为同一患者的增强CT、脑MRI、CT/MRI融合图像，GTVCT用红色线显示，GTVCT/MRI用绿色线显示。 Multiple metastases (A: Enhanced CT image; B: MRI image; C: CT/MRI fusion image). The diagrams (Fig 2 to Fig 4) of A, B, C respectively for the same patients with enhanced CT, brain MRI, CT/MRI fusion images, GTVCT with red line display, GTVCT/MRI green line display.

目前肺癌脑转移率呈升高趋势，如何更好地改善临床症状、控制局部病灶、提高影像识别的准确率以及靶区勾画的精确度是关键。本研究显示，单独应用CT勾画GTV的体积明显大于应用CT/MRI融合图像勾画GTV的体积，而主要原因是MRI较CT能够更清楚地显示肿瘤边缘的信息。通过CT/MRI图像融合技术将MRI和CT的优势结合起来，使靶区勾画相对容易，并且提高了靶区勾画的一致性，减少了主观误差。有报道^[6]^显示不同医师之间根据CT/MRI图像融合勾画的靶区，由于主观认识上的差异引起的差别小于CT靶区的差别。本研究虽未进行上述比较，但CT/MRI图像融合对于脑转移靶区勾画的一致性应该存在积极意义。目前CT/MRI融合技术尚不能解决生物靶区的勾画，因为常规MRI无法了解靶区内部的生物学信息，如乏氧、增殖等，目前PET-CT是解决上述问题的主要手段，然而PET-CT对于脑转移的成像效果不佳，无法清晰显示肿瘤边界，且存在一定的误诊及漏诊，因此，如何将CT、MRI、PET-CT的优势结合在一起或许是解决生物靶区问题的一条途径。本研究仍存在一些待解决的问题，如CT定位时虽均采用B型头枕以接近MRI扫描的体位但并不是完全一致，以及病例数较少没有针对各个病理类型进行比较，研究小组将进一步完善研究，探寻CT/MRI融合技术在临床应用的价值。
